# A Prostate Cancer Proteomics Database for SWATH-MS Based Protein Quantification

**DOI:** 10.3390/cancers13215580

**Published:** 2021-11-08

**Authors:** Ammara Muazzam, Davide Chiasserini, Janet Kelsall, Nophar Geifman, Anthony D. Whetton, Paul A. Townsend

**Affiliations:** 1 Manchester Cancer Research Centre, Division of Cancer Sciences, Faculty of Biology, School of Medical Sciences, Medicine and Health, University of Manchester, Wilmslow Road, Manchester M20 4GJ, UK; tony.whetton@manchester.ac.uk; 2 Stoller Biomarker Discovery Centre, Division of Cancer Sciences, Faculty of Biology, Medicine and Health, University of Manchester, Manchester M13 9PL, UK; janet.kelsall-2@manchester.ac.uk; 3 Department of Medicine and Surgery, Section of Physiology and Biochemistry, University of Perugia, 06132 Perugia, Italy; davide.chiasserini@unipg.it; 4 Faculty of Health and Medical Sciences, University of Surrey, Guildford GU2 7XH, UK; n.geifman@surrey.ac.uk; 5 NIHR Manchester Biomedical Research Centre and the Manchester Academic Health Sciences Centre, Manchester M13 9PL, UK; 6 Wolfson Molecular Imaging Centre, Division of Cancer Sciences, Faculty of Biology, Medicine and Health, University of Manchester, Palatine Road, Manchester M20 3LJ, UK

**Keywords:** prostate cancer, spectral library, blood proteomics, protein, peptide, mass spectrometry, SWATH-MS

## Abstract

**Simple Summary:**

Prostate cancer is the third most frequent cancer in men worldwide, with a notable increase in prevalence over the past two decades. The PSA is the only well-established protein biomarker for prostate cancer diagnosis, staging, and surveillance. It frequently leads to inaccurate diagnosis and overtreatment since it is an organ-specific biomarker rather than a tumour-specific biomarker. As a result, one of the primary goals of prostate cancer proteome research is to identify novel biomarkers that can be used with or instead of PSA, particularly in non-invasive blood samples. Thousands of peptides or assays were detected in blood samples from patients with low- to high-grade prostate cancer and healthy individuals, allowing data processing of sequential window acquisition of all theoretical mass spectra (SWATH-MS). By assisting in the detection of prostate cancer biomarkers in blood samples, this useful resource will improve our understanding of the role of proteomics in prostate cancer diagnosis and risk assessment.

**Abstract:**

Prostate cancer is the most frequent form of cancer in men, accounting for more than one-third of all cases. Current screening techniques, such as PSA testing used in conjunction with routine procedures, lead to unnecessary biopsies and the discovery of low-risk tumours, resulting in overdiagnosis. SWATH-MS is a well-established data-independent (DI) method requiring prior knowledge of targeted peptides to obtain valuable information from SWATH maps. In response to the growing need to identify and characterise protein biomarkers for prostate cancer, this study explored a spectrum source for targeted proteome analysis of blood samples. We created a comprehensive prostate cancer serum spectral library by combining data-dependent acquisition (DDA) MS raw files from 504 patients with low, intermediate, or high-grade prostate cancer and healthy controls, as well as 304 prostate cancer-related protein in silico assays. The spectral library contains 114,684 transitions, which equates to 18,479 peptides translated into 1227 proteins. The robustness and accuracy of the spectral library were assessed to boost confidence in the identification and quantification of prostate cancer-related proteins across an independent cohort, resulting in the identification of 404 proteins. This unique database can facilitate researchers to investigate prostate cancer protein biomarkers in blood samples. In the real-world use of the spectrum library for biomarker detection, using a signature of 17 proteins, a clear distinction between the validation cohort’s pre- and post-treatment groups was observed. Data are available via ProteomeXchange with identifier PXD028651.

## 1. Introduction

Prostate cancer (PCa) is the third most prevalent malignant tumour in the world, accounting for 7.3% of all cancer-related incidence rates and 3.8% of all cancer-related fatalities in 2020 [1]. The European Association of Urology (EAU-ESTRO-SIOG) [2] currently recommends analysing serum concentrations of prostate-specific antigen (PSA) with accompanying digital rectal examination (DRE) and multiparametric Magnetic Resonance Imaging (mpMRI) [3]. While PSA levels are age-stratified for diagnosis [4,5] its expression is organ-specific rather than tumour-specific and is frequently associated with non-neoplastic aetiology; likewise, DRE is insensitive [6]. Detection is typically related to unnecessary biopsies, which decide the final diagnosis by histological examination of the prostate gland. Biopsies are an invasive procedure that has an elevated risk of post-biopsy complications [7]. D’Amico categorised PCa risk stratification using PSA levels, TNM (tumour, nodes, and metastases), and histology (Gleason Score), which are widely utilised in clinical practice [8]. Nonetheless, there remains a clinical need for biomarkers to improve PCa diagnosis, patient stratification, and clinical outcome prediction.

Alternatively, new potential PCa biomarker panels are presently being investigated, and their application will ideally reshape the way clinical decision making is conducted in the future. The PHI (Prostate Health Index), PCA3 (Prostate Cancer Antigen 3) and Prolaris tests, for example, have been approved by the US Food and Drug Administration (FDA), whereas the Clinical Laboratory Improvement Amendments (CLIA) have accepted the 4Kscore panel algorithm, Mi-Prostate score (MiPS), OncotypeDx, ConfirmMDx, and Decipher [3,9]. The majority of the time, they are used in combination for clinical decision making about initial and repeated biopsies as well as monitoring therapy outcomes; nevertheless, no single biomarker appears to be superior to PSA.

Traditionally, clinical diagnostics relied heavily on antibody-based detection techniques, which had limitations. There has recently been a considerable amount of evidence of study in biomarker identification using high throughput technology. Mass spectrometry (MS) is a powerful tool that allows for a thorough understanding of the proteome aberrations of the complex biological matrix with disease development. It has been extensively used for the development of new biomarkers in a variety of cancer types [10,11,12,13]. Although genomics and transcriptomics analyses have potential, not all genetic abnormalities are readily converted into disease phenotypes [14]. Conversely, protein molecules are appropriate representations of disease progression and serve as active targets for cancer therapeutics [15].

SWATH-MS (sequential window acquisition of all theoretical fragment-ion spectra mass spectrometry) is a data-independent acquisition (DIA) approach for ionising and fragmenting all peptides in a biological sample. The precursor and product ions are separated by an orderly and impartial series of predefined precursor isolation windows [16]. All precursor and product ions data will be permanently logged in a single scan within the specified mass range, requiring a targeted data extraction approach. This technique is based on a sample-specific spectral reference library containing all the peptides obtained from DIA data. As a result, reference libraries are essential to the success of SWATH-MS studies [17].

Here, we offer a bespoke spectral library for SWATH-MS based measurement of circulatory proteins produced in the blood of PCa patients to aid and improve PCa blood-based biomarker discovery.

## 2. Results

### 2.1. Characteristics of the Study Participants

**Figure 1:**
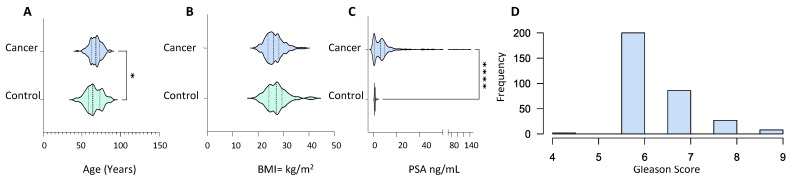
Demographic and clinical characteristics of PCa patients (*n* = 373) and healthy male volunteers (*n* = 134) used for spectral library generation. (**A**) Age distribution; (**B**) body mass index; (**C**) prostate-specific antigen (in ng/mL); and (**D**) Gleason score frequency distribution. All characteristics were captured at the time of sample collection. Statistical significance was determined by a non-parametric Mann–Witney test. *p* value shown as: 0.01–0.05 (*) and <0.001 (****).

Serum samples from 373 PCa patients with abnormal prostate on digital rectal examination, symptomatic presentation with high PSA levels and abnormal biopsies, or patients with a significant rise in PSA associated with urinary symptoms were used to create the library (Table S1). Serum samples were also taken from 134 healthy control subjects with normal DRE and PSA levels of less than 1 ng/mL (1 ng/mL). The cancer group was older (*p* value = 0.01) with an average age of 67.57 ± 7.48 compared to 65.48 ± 10.22 in the control group (Figure 1A). The BMI of both groups lay within the same range with no statistical difference, 26.37 ± 3.48 and 27.19 ± 4.24 for the cancer and control group, respectively (Figure 1B). PSA levels in the cancer patients were significantly higher than in the control group (*p* value ≤ 0.0001) with mean PSA of 13.19 ± 93.52 and 0.97 ± 0.514, respectively (Figure 1C). The majority of patients in the cancer group presented with a Gleason score of 6, whereas the number of patients with a Gleason score of 7, 8 and 9 is small (Figure 1D). The majority of the PCa patients exhibited T1 and T2 staged tumours with occasional cases of T3 stage along with nodal invasion and metastasis (Table S1).

### 2.2. The Spectral Libraries

The in silico spectral library contained 41,995 transitions, 4503 peptides, and 346 proteins. The consensus library of serum PCa built on the Lumos instrument contained 83,346 transitions, 9577 peptides, and 760 proteins, whereas the consensus library built on the Sciex-TOF 6600 in micro-flow and nano-flow contained 50,160 transitions, 6210 peptides, 484 proteins, and 36,624 transitions, 4478 peptides, and 712 proteins, respectively. The incorporation of all consensuses and in silico libraries into a comprehensive PCa serum library yielded 114,684 transitions, 13,793 peptides, and 1227 unique proteins. Table 1 displays the assay statistics for each acquired library.

**Figure 2:**
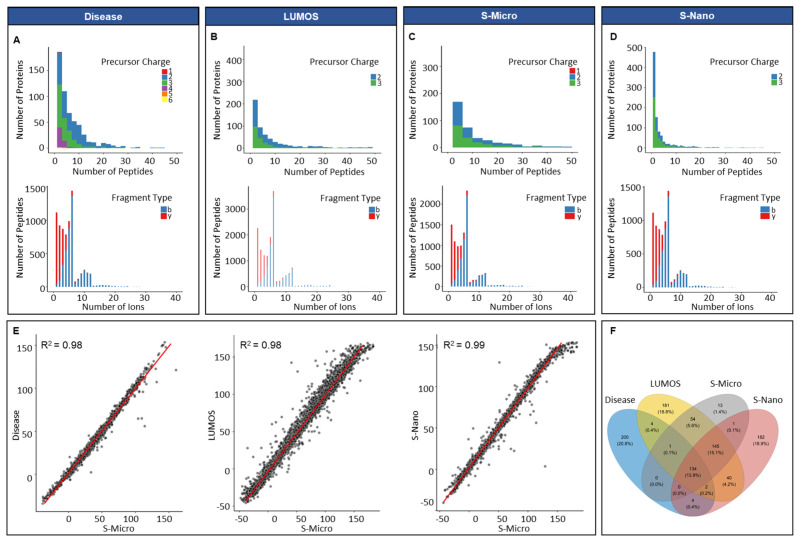
Overview of PCa spectral libraries generated in silico and built in-house. Frequency distribution of peptides per protein and ion per peptides of (**A**) in silico library (Disease); (**B**) in-house library built on Orbitrap Fusion Lumos Tribrid Thermo Scientific (Waltham, MA, USA) mass spectrometer (in micro-flow mode); (**C**) in-house library built on Sciex TripleTOF 6600 mass spectrometer (in micro-flow mode, S-Micro); (**D**) in-house library built on Sciex TripleTOF 6600 mass spectrometer (in nano-flow mode, S-Nano); (**E**) retention time (RT) correlation between the four libraries by using S-micro as a base library; (**F**) Venn diagram presenting the proteotypic proteins overlap between the four libraries.

The average number of peptides per protein in the in silico disease-associated library was six, and the average length of peptides was 15 amino acids (Figure S2). A minor percentage of the precursor ions (0.07%, 30) was singly charged. Approximately 54.6% (22,944) of precursor ions was doubly charged, 35.1% (14,760) had a charge of 3, 8.2% (3450) had a charge of 4, 1.4% (666) had a charge of 5, and 0.3% (132) had a charge of 6. All proteins included were proteotypic; hence, the existence of shared proteins was not observed (Figure 2A). In respect of the distribution of fragment, types that presented y-ions were more frequently observed (82%, 34,572) compared to b-ions (18%, 7422) (Figure 2A). The average number of peptides per protein for Lumos library was approximately nine, with an average length per peptide of 15 amino acids. The total number of proteotypic proteins was 561 and shared proteins was 199 (Table S2). The percentage of double and triple charged precursor ions was 63% (52,722) and 37% (30,624), respectively, whereas the percentage of y-ions was predominant at 83% of total ions (69,153) compared to b-ions (17%, 14,193) (Figure 2B). For the S-nano library, the frequency distribution of precursor charge was doubly charged at 41% (or 15,012), triply charged at 59% (or 21,612 in total), and ion type was: y-ions; 81% or 29,524 in total; b-ions, 19% (or about 7100 in total), similar to the generated Lumos library (Figure 2D). The average number of peptides per protein was five, with a mean count of 15 amino acids per protein. The total count of proteotypic and shared proteins was 508 and 204, respectively. The library acquired on Sciex TOF 6600 instrument on micro-flow mode resulted in the lowest number of total proteins identified 484; out of them 136 were shared. The average peptides identified per protein were eight with a mean length of 15 amino acids and precursor charge distribution of 0.01% (6), 52% (26,196) and 48% (23,958) for the single, double, and triple charge, respectively (Figure 2C). In parallel to other spectral libraries, y-ions (82%, 40,883) were also predominant in the S-micro library compared to b-ions (18%, 9277) which were observed in a smaller amount (Figure 2C). The precursor ion m/z ranged for samples run on Sciex-TOF 6600, and Lumos was between 399 to 1249 m/z, which corresponds well to the MS mass range settings for the full coverage of peptides through SWATH analysis (Figure S2). The product ion m/z ranged between 350–1499 m/z for samples run on Sciex (in micro-flow and nano-flow), which went between 350–1998 m/z for samples run on Lumos (Figure S2).

For integrating all in-house built libraries into a comprehensive PCa serum library, retention time correlation was determined by taking S-micro as the base library. The reason behind the selection of S-micro as base library was the similarity between the MS settings for the data acquisition for DDA and the SWATH-MS analysis. The retention time correlation (R^2^) between S-micro with Disease, Lumos and S-nano library was 0.98 (Figure 2E). The overlap between proteotypic proteins of the four libraries showed 200 proteins unique to disease library, 182 unique to S-nano, 181 unique to Lumos library and 13 unique to S-micro (Figure 2F, and Table S2). Overall, acquisition of data at micro-flow coupled with DDA offered the lowest amount of exclusivity and coverage compared to nano-flow coupled DDA mode.

### 2.3. Integrated PCa Serum Spectral Library

**Figure 3:**
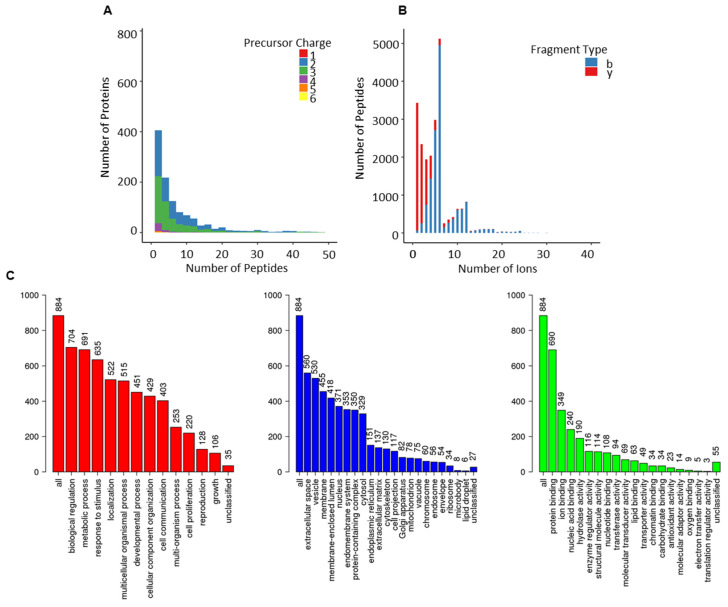
Overview of combined-PCa serum spectral library. (**A**) Frequency distribution of peptides per protein; (**B**) ion per peptides; (**C**) gene ontological analyses of the proteotypic proteins from combined-PCa library mapped using the WebGestalt Webserver. The biological processes, cellular processes and molecular function categories are shown, where the height of each bar represents the percentage of mapped IDs per category.

Assay statistics for the combined library are given in Table 1, and full characterisation is presented in Figure 3. The precursor charge distribution showed the abundance of precursor charge 2 (55%, 63,618) followed by precursor charge 3 (41%, 47,448; Figure 3A). The remaining charged species (charge 1 = 0.03%, 36; charge 4 = 2.5, 28,989; charge 5 = 0.6%, 624; charge 6 = 0.11, 132) were less prevalent. Similarly, y-ions (83%, 95,630) were predominant compared to b-ions (17%, 19,138; Figure 3B). The precursor m/z charge ranged between 399–1248 m/z and the product charge between 350–1873 m/z (Figure S2). The frequency distribution of peptides per protein presented an average of 7 peptides per protein, and the mean amino acid count per peptide was 15 (Figure S2). The total protein count comprised 1227 (950 were proteotypic; Table S2).

The use of gene ontology to classify proteotypic proteins revealed that the majority of proteins are present in extracellular areas and that their biological and molecular roles are abundant in regulatory, structural, and binding properties, influencing cellular and metabolic processes. (Figure 3C).

**Figure 4:**
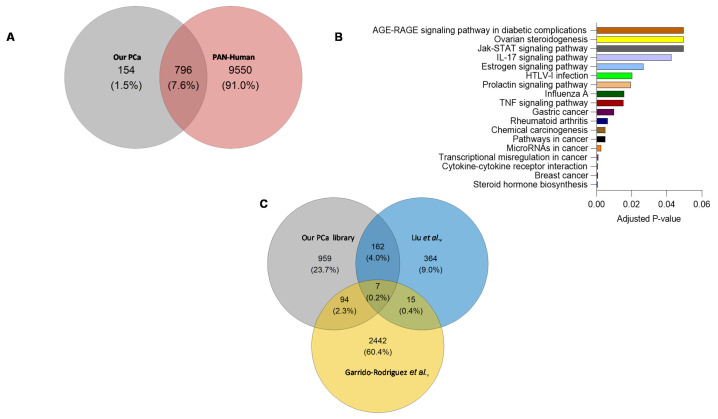
(**A**) Overlap between proteotypic proteins in the combined-PCa and Pan-Human spectral libraries. (**B**) KEGG pathway analysis of proteins unique to combined-PCa spectral library. (**C**) Overlap of the total protein count of our combined serum PCa spectral library and PCa spectral libraries previously published from PCa tissue and cell lines samples [19,20].

A comparison of proteotypic proteins in the combined-PCa library with the Pan-Human library [18] (one of the most comprehensive spectral libraries covering over 10,000 human proteins), revealed 154 proteins unique to the combined-PCa library (Figure 4A) that were enriched in cancer and hormonal signalling pathways (Figure 4B). These proteins include the previously identified PCa marker KLK isoforms (KLK2, KLK11) as well as the products of additional PCa-related genetic markers such as TNF, MYC, BRCA2, and ERG1. Tissue origin in serum proteome studies has not in general provided clear indication of organ involvement. Nevertheless, we examined this using the Human Protein Atlas and the 154 unique protein list. Of these proteins, KLK2, TMEFF2, SPDEF, GPRC6A, ANO7, ALOX15B, SLC45A3, PAGE4 and TRPM8 are prostate-specific, while PTGS2 is ductus deferens and seminal vesicle specific, BRCA2, GGN, ZNF233, CAGE1, HSD17B3, ZSCAN5B, GAGE2A, PHOX2A, ARGFX, ESR2, DBX1, ART3, TERT and SHC4 are testis-specific. Organ associations for the complete list of 154 proteins identified using the combined PCa library is shown in Table S3. In Figure S3, we presented the protein-protein interactions of 154 different entities. Furthermore, the total protein content of the combined-PCa serum library was compared to other PCa-related libraries obtained from the literature (built on different sample types) (Figure 4C). Liu et al. identified 548 proteins in a spectrum library with shotgun measurements of 22 non-aggressive (Gleason score 5–7) and 11 aggressive (Gleason score 8–9) pooled prostate tissue samples obtained from PCa patients after surgery [19]. Garrido-Rodriguez et al. discovered 2558 proteins by using pooled samples of human PCa cell lines DU145, LNCaP, PC-3, and 22Rv1 to generate a spectral library through a shotgun approach [20]. When these two libraries were compared to our combined library, we discovered a total of 959 distinct proteins in our combined-PCa serum library (Table S4). The cell-lines library found 2442 different proteins, but the prostate tissue library discovered the fewest (364). Our combined-PCa collection has reported the most PCa-related circulatory proteins and the most comprehensive coverage of the PCa blood proteome, to the best of our knowledge.

Furthermore, 1% of all human gene mutations have been linked to cancer development. Germline mutations account for 20% of all mutations, with somatic mutations accounting for the remainder [21]. To identify proteins associated with tumours or tumorigenesis, we compared a list of 569 genes from the catalogue of somatic mutations in the cancer (COSMIC) database acquired from the Human Protein Atlas to a list of genes (acquired from Uniprot IDs) from our combined library to assess if there was any overlap. We discovered 64 genes that were shared by both sources, yet 886 were unique to the combined PCa library and 499 were unique to the COSMIC database (Table S5).

### 2.4. Spectral Library Validation

An independent cohort of PCa patients’ serum samples was used for library validation. The patient cohort included pre-treated patients (*n* = 29) with rising PSA levels who needed treatment, as well as patients who had undergone treatment (*n* = 14) who received brachytherapy/radiotherapy (T1 and T2 staged patients), external beam radiotherapy when diagnosed with urinary complications (T3 staged patients; Table S6). The average difference in PSA before and after treatment was 11.91 ± 24.68 and 5.47 ± 6.79, respectively.

**Figure 5:**
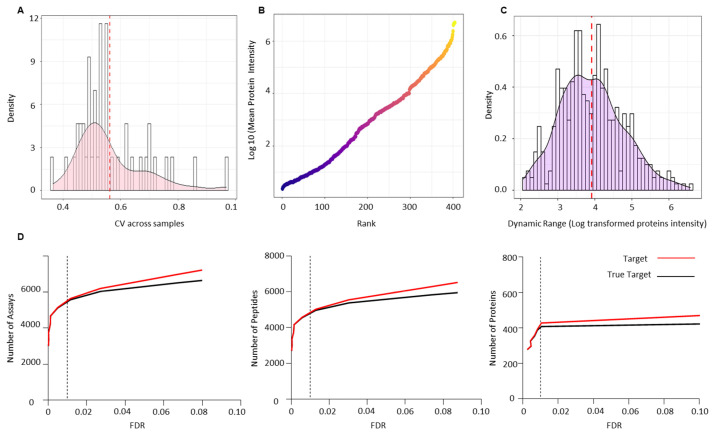
Technical validation of serum PCa spectral library using an independent PCa patient cohort of pre- (*n* = 29) and post-radiotherapy (*n* = 14) patients. (**A**) Coefficient of variance (CV) across all the validation samples. (**B**) Dynamic range of the PCa serum proteome based on the rank by abundance of the log10 mean intensity of all identified proteins in the validation cohort. (**C**) Dynamic ranges of individual proteins detected in the validation cohort based on the ratio of the maximum and minimum log10 protein intensities. (**D**) Number of transitions, peptides and proteins identified at 1% FDR across the validation cohort.

Raw data files from the validation cohort were analysed against the combined-PCa serum spectral library. CV was calculated to assess the technical variation across all biological samples used for validation and was found to be less than 10, which is consistent with the possibility of technical variation (Figure 5A). One of the most challenging issues in quantifying the serum proteome is the dynamic range of proteins present, which makes measurement difficult. Since the serum proteome is usually occupied by highly abundant proteins, measuring low abundance proteins is difficult. Immune depletion of the key abundant proteins is one approach to addressing this issue. We report the dynamic range of the PCa serum proteome after depleting the 12 most abundant proteins and ranking all 404 identified proteins in the validation cohort by their log mean intensity based on their abundance (Figure 5B). The PCa proteome is six orders of magnitude higher than the typical dynamic range of serum or plasma reported in the literature (this lies within 2–4 log range). The dynamic ranges of individual proteins detected in the PCa serum validation cohort were then calculated using the ratio of the maximum and minimum log10 protein intensities (Figure 5C). The identified proteins had dynamic range values ranging from 2.07 to 6.57 (SD = 0.85) orders of magnitude. True targets were estimated using a target-decoy approach, which identified 5572 transitions, 4953 peptides, and 404 proteins with an FDR of 1% (Figure 5D) (Table S7). Table S8 shows the sum of transitions, peptides, and proteins across each sample from the validation cohort.

**Figure 6:**
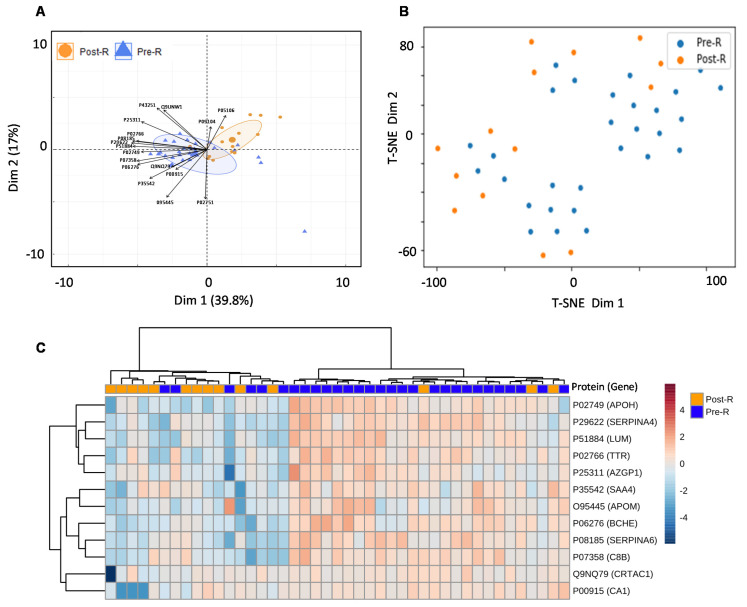
Usability of the combined PCa serum library. (**A**) PCA showing separation between pre- and post-radiotherapy samples of validation cohort after feature selection by random forest. (**B**) t-SNE plot showing separation between pre- and post-radiotherapy samples in the validation cohort after feature selection by random forest. (**C**) Heatmap showing the clustering of pre- and post-radiotherapy samples based on significant (*p* value ≤ 0.05) and non-variable proteins. Each coloured cell on the map represents a data point’s concentration value, with samples arranged in rows and features arranged in columns. Distance was calculated using the “Euclidean” approach, while clustering was performed using the “ward” method. The Uniprot accession number of each given protein are as follows: APOM (O95445), TTR (P02766), APOH (P02749), SAA4 (P35542), CA1 (P00915), ITGB3 (P05106), SERPINA6 (P08185), BCHE (P06276), SERPINA4 (P29622), C8B (P07358), LUM (P51884), CRTAC1 (Q9NQ79), AZGP1 (P25311), FN1 (P02751), ENO2 (P09104), MINPP1 (Q9UNW1) and BTD (P43251).

For the real-world application of the spectrum library in biomarker identification, the separation between the validation cohort’s pre- and post-treatment groups was assessed using a random forest model after elucidating the significant entities demonstrating discrimination between the two groups. This resulted in a list of 17 proteins including; APOM (*p* value = 0.00080582), TTR (*p* value = 0.0010406), APOH (*p* value = 0.001424), SAA4 (*p* value = 0.0026501), CA1 (*p* value = 0.0054433), ITGB3 (*p* value = 0.0054809), SERPINA6 (*p* value = 0.007307), BCHE (*p* value = 0.0078392), SERPINA4 (*p* value = 0.019909), C8B (*p* value = 0.030581), LUM (*p* value = 0.031786), CRTAC1 (*p* value = 0.038456), AZGP1 (*p* value = 0.047812), FN1 (*p* value = 0.15521), ENO2 (*p* value = 0.21444), MINPP1 (*p* value = 0.7663) and BTD (*p* value = 0.98711), indicating that the pre- and post-radiotherapy samples were well separated (Figure 6A,B, Table S9). To improve the separation of the pre- and post-radiotherapy samples, highly variable proteins (ITGB3) and proteins with non-significant *p* values (FN1, ENO2, MINPP1, and BTD) were removed from the heatmap. This further demonstrated that the pre- and post-radiotherapy samples clustered differently based on the selected proteins (Figure 6C).

**Figure 7:**
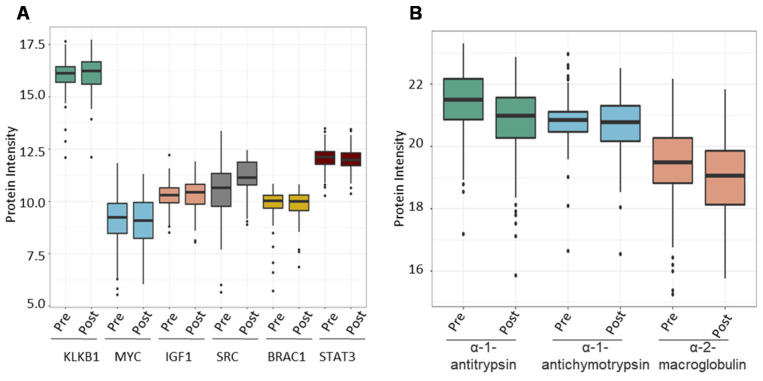
The combined PCa serum library’s utility in finding known prostate cancer-associated proteins. (**A**) Expression of known prostate cancer biomarkers across samples from the validation cohort. (**B**) Expression of proteins that bind to free PSA in human serum.

Following that, we investigated the ability of the spectral library to quantify six PCa biomarker candidates previously reported in the literature across the validation cohort. We chose plasma kallikrein (KLKB1), Myc proto-oncogene protein (MYC), insulin-like growth factor I (IGF1), Src proto-oncogene tyrosine-protein kinase Src (SRC), breast cancer type 1 susceptibility protein (BRCA1), and signal transducer and activator of transcription 3 (STAT3). PSA (KLK3, a widely used clinical biomarker for PCa) was shown to dramatically boost the expression of KLKB1 (average intensity = 16.05) serine proteases that demonstrate unregulated expression in PCa. Other biomarkers were overexpressed as well (MYC = 8.5, IGF1 = 10.28, SRC = 10.75, BRAC1 = 9.78, STAT3 = 12.05), albeit not as significantly as KLKB1 (Figure 7A). Finally, we wanted to evaluate the amount of expression of proteins that bind to PSA in order to demonstrate the reference library’s additional applications. PSA attaches to one of three proteins found in serum: alpha-1 antitrypsin, alpha-1 antichymotrypsin, and alpha-2 macroglobulin. We determined the mean expression of these PSA binding proteins throughout our validation cohort samples to be 21.22, 20.78, and 19.34 for alpha-1 antitrypsin, alpha-1 antichymotrypsin, and alpha-2 macroglobulin, respectively (Figure 7B).

## 3. Discussion

Blood is a highly complex bodily fluid composed of cellular and biochemical components separated from serum after removing the cells and clotting factors. Human serum is an easily accessible, non-invasive source for biomarkers and the proteome within presents a profile of proteins originating from normal and damaged cells, tissues, and organs. While the dynamic levels of these circulatory proteins may correlate with an individual pathophysiological state, the concentrations of these can span a 10 order of magnitude, making the profiling of these difficult when using modern LC-MS/MS methods [22]. Because the most abundant proteins in blood account for 99% of total protein content, reducing them would increase the dynamic range of the serum and increase the visibility of the less abundant proteins, which are more likely to serve as markers and accurate indicators of an individual’s physiological status [23].

To increase the signal-to-noise ratio when building bespoke spectral libraries from clinical samples several strategies can be employed. First, one may be able to adopt a pooling strategy, grouping the samples in sub-pools to increase the probability to detect low abundance proteins or putative “sample-specific proteins” as our group has previously undertaken in other studies [24]. However, this current study has been performed in serum samples and not in proximal fluids. The latter may have a more diverse proteome, or more precisely, a proteome enriched in proteins derived from specific organs. For serum samples we adopted an alternative strategy relying on a deep fractionation of the samples and analysis on three different instruments. Using a deep fractionation will help to overcome the major hurdle of serum proteomics, which is the presence of few, yet highly abundant proteins (albumin, globulins, inflammation-related proteins) [25]. Additionally, the use of different instrumentation and set-ups will increase the ion diversity of the spectral library, in turn increasing the number of peptides and proteins. The combination of the spectral libraries obtained from the Orbitrap and Q-TOF instruments showed a much larger number of consensus proteins eventually included in the combined PCa serum library.

The emphasis in PCa clinical research has shifted away from single biomarker analysis and toward the identification of panels of biomarkers [26]. Researchers are constantly striving to create novel proteome biomarkers in non-invasive biological matrices for improved diagnosis and risk classification. The detection of low abundance proteins using new and powerful MS platforms in conjunction with depletion techniques to offer full coverage of proteome aberrations in response to disease states has been a significant achievement in proteomics. SWATH-MS is one such tool that enables the comprehensive and permanent storing of information about peptides and their fragmented species within a specific m/z range [16]. Prior knowledge in a reference spectral library is necessary to extract data from permanent digital maps utilising a peptide-centric technique [17]. We present a complete online database of 1227 proteins associated with serum samples from PCa patients and healthy individuals (controls) to facilitate PCa blood-based proteomics for clinical research (Table 1, Figure 3).

This study comprehensively covers the serum proteome matrix by utilising a variety of LC workflows (nano and micro-flow) and instrument types (Table 1, Figure 2). Microflow separations are commonly performed at flow rates of less than 100 μL/min [27], whereas nano-flow separations are accomplished at flow rates as low as a few nL/min [28]. The normalised retention time added dimensionless value to both LC setups employing iRT peptides. Comparing the two workflows on the Triple TOF 6600 revealed a significant variation in the overall number of proteins detected, which was 484 and 712, respectively. Micro-flow discovered 68 unique proteotypic proteins, and nano-flow identified 228 unique proteotypic proteins. This reveals that a nano-LC workflow covers the biological matrix more thoroughly than a micro-flow approach on a similar device. However, micro-flow can avoid time-consuming column blockage and can maximise sample throughput, as a result, it is the preferred approach in clinical proteomics. It has already been established that a spectrum library constructed using TripleTOF data identifies fewer proteins than one constructed using Orbitrap data [29]. The comparison of nano-LC workflows on TOF 6600 and Orbitrap also revealed an increase in the total number of proteins identified (*n* = 760) in the latter.

The clinical characteristics of the PCa patients included in this study ranged from low-risk indolent cancer to high-risk nodal invasion and metastasis (Table S1, Figure 1), allowing for the establishment of a comprehensive blood proteomic database for extensive PCa research in early and late staged PCa. SWATH reference libraries previously created for the assessment of PCa samples focussed on tissues and cell lines [19,30,31,32]. However, a major hurdle in using biomarkers based on tissue is whether this will result in a non-invasive clinical diagnosis [7] and whether the cell lines are real representations of cancer which are modelling [33]. As a result, blood and other readily accessible biofluids have become the future of biomarker research. In a study using PCa serum samples, the researchers created a pan-human plasma spectrum library that contained 1151 plasma glycoproteins originating from five solid tumours, including PCa [34]. To the best of our knowledge, the library presented here is the first dedicated spectral library to analyse PCa blood proteome.

The PCa serum spectral library contains a diverse collection of extracellular proteins involved in metabolic, physiological, developmental, and signalling activities (Figure 3). Comparing our PCa-serum spectrum library to the pan-human spectral library [18], a comprehensive proteome database of plasma, tissue, and cell line samples, revealed 154 proteins unique to our PCa-serum collection (Figure 4). Among these are proteins associated with carcinogenesis, enriched in cancer-related signalling pathways, and proteins involved in hormone signalling. TNF [35], BRCA2 [36], MYC [37], and IL6 [38] are some examples of proteins that are frequently associated with PCa and are also known to function as biomarkers for other types of cancer. While comparing the total protein content of our PCa serum library to that of other PCa-related tissue and cell line reference libraries revealed 959 proteins unique to serum samples. This demonstrates the critical nature of having a sample-specific reference library for extending the coverage of information in SWATH maps and enhancing the clinical utility of SWATH-MS in blood-based proteomics of PCa.

Our strategy was to include a large number of patients in the cohort used to construct the library in order to increase the diversity and heterogeneity of the clinical samples included. As such, 75% of all patients enrolled for this study were comprised in the discovery cohort for library building. An independent cohort of pre-radiation and post-radiation PCa patients was used to determine the utility and technical validity of the PCa-serum spectral library. A total of 404 proteins in the SWATH maps with a 1% FDR have been identified, with a good dynamic range and coverage compared to other relevant studies, which found 272 and 249 proteins on SWATH blood proteome tests of five solid carcinomas [34] and pulmonary cancer [39] respectively. Separation across clinical conditions was also evident using the top significant proteins, which increase the clinical utility of the spectral library in real-world experiments (Figure 6). Our rationale for including different patients in the validation cohort was to keep this validation independent from the discovery cohort. Testing on all available samples may result in overestimation of the library’s performance. By evaluating the performance of our library blindly in an independent group of samples, we established the first step toward analysis of larger cohorts of patients. As we have now established a robust, bespoke library for the community we will perform validation analyses as other cohorts become available.

The KLKB1 gene codes for the production of a protein known as pre-kallikrein that is produced in the liver and circulates in the blood and is involved in blood clotting [40]. MYC mRNA and protein overexpression in PCa tumour foci corresponds with disease severity [37]. IGF1, a downstream MAPK and PI3K receptor, boosts their signalling and promotes the formation of PCa [41]. The recurrence of castration-resistant PCa is influenced by androgen receptor signalling [42]. BRCA genes have been linked to breast and ovarian cancer. They also raise the risk factors for aggressive PCa in men because cells are less capable of repairing DNA damage without BRCA genes, making them more vulnerable to tumour formation [43]. Similarly, STAT3 and its upstream activator, the IL6 receptor, have been linked to PCa development and may represent future therapeutic targets [44]. We confirmed the expression of PCa-related proteins across SWATH maps of validation cohort samples to establish the utility of our reference library for biomarker research (Figure 7A). PSA enters the bloodstream free or bound to one of three serum proteins: α-2 macroglobulin, α-1-antichymotrypsin and α-1antitrypsin. PSA appears to be abundant in bound form in PCa males, indicating that PCa cancer cells produce more of these binding proteins [45]. We validated that they were included in the PCa validation set (Figure 7B). 

This study shows a comprehensive range of PCa serum peptide assays and may aid in quantifying low, moderate, and high-grade PCa. Improved blood-based biomarkers are of critical clinical importance to provide improved, non-invasive diagnoses that will likely replace PSA. The novel TMPRSS2-ERG gene mRNA and PCA3 genetic marker are already being utilised in clinical practice, but it is unclear if the resulting protein alterations affect disease progression and treatment response [26]. As a result, proteome research is still required for biomarker identification to be possible. While a SWATH-MS-based analysis may allow for rapid differentiation of cancer and non-cancer samples, it is unlikely to be easily converted into routine clinical practise. However, it may pave the way for developing clinically manageable immunoassay-based technologies.

## 4. Materials and Methods

### 4.1. Research Ethics and Approval

This study was approved by Yorkshire and the Humber-Leeds East Research Ethics Committee under reference no. 08/H1306/115+5 and IRAS project ID 3582. All study participants submitted written consent prior to the collection of blood samples. All personal data pertaining to participants were kept strictly confidential and processed in accordance with the Data Protection Act (1998). The inclusion criteria for samples from PCa patients are abnormal DRE, rising PSA levels, symptomatic patients with urinary symptoms, and histological confirmation of PCa, while the criteria for their matched controls are normal DRE, PSA 1ng/mL, asymptomatic individuals with no urinary symptoms, and no detection of PCa on histological analysis.

### 4.2. Sample Collection

**Figure 8:**
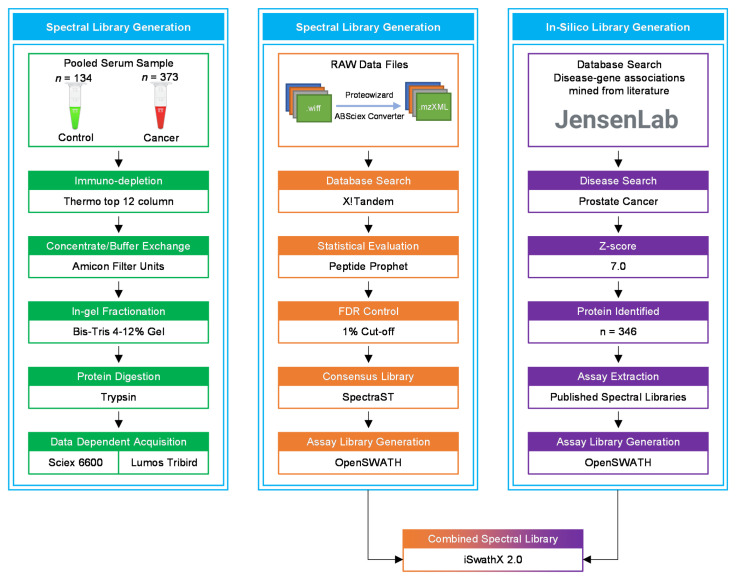
A flowchart of the data acquisition and spectral library generation workflow. The pooled samples were immuno-depleted of the top 12 abundant proteins, followed by protein concentration, in-gel fractionation, and in-gel tryptic digestion. All samples were analysed using LC-MS/MS for data dependent acquisition (DDA). In the OpenSWATH library generation pipeline, X!Tandem was utilised for database search of raw MS files. The in silico library was created by extracting PCa-related proteins from the Disease database using a z-score cut-off of 5.0. iSwathX, a web-based tool, was used to merge all four assay libraries.

Peripheral blood was collected into red-capped BD Vacutainer® blood collection tubes (BD Biosciences, Wokingham, UK) from 373 newly diagnosed PCa patients and 134 healthy male individuals, used as controls. Vacutainers containing blood were inverted five times and incubated at room temperature for 30 min before centrifugation at 3000 rcf (g) for 10 min. The clear fraction of serum was further stored at −80 °C until analysis. All samples were processed within 2 hours of collection. The serum samples from PCa patients and healthy controls were pooled separately for library generation (Figure 8).

### 4.3. Sample Preparation

To maximise the depth of proteome penetration, pooled PCa and healthy control samples were processed using Pierce^TM^ top 12 abundant protein depletion spin columns following the manufacturer’s recommendations (Thermo Fisher Scientific, Loughborough, UK. Concentration and purification of depleted serum samples were performed using Amicon® ultra-0.5 centrifugal filter devices (Cat #: UFC5003BK, Sigma-Aldrich, Merck KGaA, Darmstadt, Germany). To reduce the salt concentration in the serum samples, they were subjected to a buffer exchange with 25 mM ammonium bicarbonate (Sigma-Aldrich, Merck KGaA, Darmstadt, Germany). The total protein content of concentrated serum samples was quantified using the bicinchoninic acid assay (BCA test, Thermo Fisher Scientific, Loughborough, UK). The total protein concentration was then normalised to 60 μg per 140 μL of sample using 25 mM ammonium bicarbonate, 35 μL LDSx4 (Cat #: NP0007, Invitrogen, Thermo Fisher Scientific, Loughborough, UK), 10 mM dithiothreitol (Cat #: 17-1318-02, GE HealthCare Life Sciences, Amersham, UK), and 1% sodium deoxycholate (Cat #: 75746-250g, Sigma-Aldrich, Merck KGaA, Darmstadt, Germany) and denatured for 2 min at 95 °C. Cell lysates were cooled at room temperature. Proteins were alkylated by adding 750 mM iodoacetamide (Cat #: I1149-25g, Sigma-Aldrich, Merck KGaA, Darmstadt, Germany) (Final concentration 50 mM) to a final volume of 150 µL and incubating at room temperature in the dark for 20 min with intermittent mixing.

### 4.4. In-Gel Fractionation

Gel fractionation was performed by putting 70 µL of reaction mix per well into a 10-well Bolt NuPAGE 4–12% Bis-Tris gel (Invitrogen, Cat #: NW04120BOX) and running at 200 Volts for 22 min in MES Buffer. It was soaked in Instant Blue (Sigma, Darmstadt, Germany, Cat #: 1SB1L) dye for 15 min to stain the gel. The gel was divided horizontally into ten equal-sized strips (Figure S1) and then vertically, with the lanes of each protein sample serving as a guide. Using a clean scalpel, the gel slices were then transferred to the wells of a 96-well plate. To sufficiently destain, the pieces were repeatedly washed with 50 mM ammonium bicarbonate and 50 mM ammonium bicarbonate/50% acetonitrile. Each washing cycle took 10 min on a shaking rotator. After the final wash, the gel pieces were dried for 10 min at 50 °C in a centrifugal evaporator.

### 4.5. In-Gel Tryptic Digestion 

Gel fragments were submerged in 150 µL of 50 mM ammonium bicarbonate/10% (*v*/*v*) acetonitrile containing 5 ng/µL trypsin and centrifuged (1000× *g*) for 1 min at 4 °C, followed by 30 min on ice. To ensure that all gel components were entirely immersed, wells were topped up with 50 mM ammonium bicarbonate and incubated overnight at 37 °C. The tryptic peptides were extracted by repeatedly adding 10% (*v*/*v*) formic acid in acetonitrile to each sample. Each wash was followed by the transfer of supernatant containing peptides to a non-Lobind 96-well plate and vacuum drying at 55 °C. Dried peptides were kept at −20 °C until further analysis was required.

### 4.6. Data Dependent Acquisition (DDA) Analysis

To generate the library, dried peptides were reconstituted in 5% acetronitrile/0.1% formic acid, 10 fmol/µL Pep-CalMix (Cat #: 5045751, A B Sciex, Warrington, UK), and 10× iRT (Biognosys AG, Schlieren, Switzerland, in a 50:1 ratio). The LC-MS was loaded with reconstituted samples for nano- and micro-flow DDA analysis. Nano analysis (Eksigent, Dublin, CA, USA) was performed using an Acquity uPLC M-Class, C-18 column (Waters, Part No: 186007484) with a flow rate of 300 nL/min and a runtime of 87 min. Microanalysis was performed using an analytical column—YMC-Triart C18 column (YMC Europe GmbH, Dinslaken, Germany, S/N 17019) with a flow rate of 5 µL/min and a run time of 87 min. The LC gradients for nano- and micro-flows were composed of 0.1% formic acid/LC-MS grade water and 0.1% formic acid/acetonitrile. DDA analysis was carried out on a Sciex Triple TOF 6600 Mass Spectrometer (AB Sciex, Warrington, UK) using a nano-flow source with a 5 µL injection volume and a 300 nL/min flow rate for nano-flow analysis and a microflow duospray source with a 10 µL injection volume and a 5 µL/min flow rate for microflow analysis. For both analyses, the TOF-MS mass range was 400–1250 Da, and the product ion mass range was 100–1500 Da [46]. Additionally, 5 µL (~1 μg) of each reconstituted sample was loaded on a 10 cm fused silica AcclaimTM PepMapTM 5 μm 100 Nano-Trap Column (Thermo Fisher Scientific, Loughborough, UK) using a U3000 RSLC high-pressure nanoLC (Dionex, Cambereley, UK) as described by Njoku et al. Eluting peptides were quantified using a Thermo Scientific Orbitrap Fusion Lumos Tribrid mass spectrometer operating in DDA mode [24].

### 4.7. Building the SWATH Spectral Library

All LC-MS/MS raw data (.wiff/.wiff.scan) files obtained from the Sciex Triple TOF 6600 Mass Spectrometer were searched against the UniProtKB/Swiss-Prot database of 20,271 reviewed, non-redundant protein species (without isoforms) (Accessed 17 December 2015), supplemented with iRT peptides, and decoy sequences using X!Tandem (Version PILEDRIVER—1 April 2015). Before searching against the database, the raw data (.raw) files collected from the Orbitrap Fusion Lumos Tribrid mass spectrometer were converted to the mzXML format and centroided. The database search was performed using the default X!Tandem parameters [17] with few adjustments as described in Njoku et al., 2021. To generate the libraries, SpectraST was used in conjunction with the OpenSWATH workflow to obtain the consensus libraries in .TSV format. The FDR of each assay library was adjusted to 1% using a target-decoy database. To facilitate use of our spectral library by other researchers, we utilised relatively standard OpenSWATH settings and included only b and y ions which were generally the most intense and a minimum of 6 fragment ions per peptide. We limited the maximum number of ions per peptide to 6 in order to extract the most intense transitions for inclusion in the SWATH assay library (Code: spectrast2tsv.py -l 350,2000 -s b,y -x 1,2 -o 6 -n 6 -p 0.05 -d -e -w ). This setting permits the incorporation of all y and b ion series especially y10 or y11, but only if they are particularly intense and observable. Previous investigations in our laboratory have demonstrated that these settings have no effect on quantification when a larger number of less intense ions are included [24]. To generate the in silico library, a list of proteins associated with PCa was collected from the Jensen lab’s “Disease database” [47], using a z-score cut-off of 5.0. For the list of PCa-related proteins, information regarding their peptides and fragments was raised via a synthetic human proteome database [48] and/or repository of 10,000 human proteins [18]. Using the default parameters, iSwathX (version 2.0) [49] was utilised to create an extended reference library by integrating all four bespoke spectral libraries. The base library was collected on a Sciex instrument in micro-flow mode, and the remaining libraries were added by combining the protein accession numbers and deleting any redundancy. The following information was added: “transition name” and “transition group id” to the combined library using a R script (R version 4.0.2). The combined consensus library’s .TSV format was converted to Transition Markup Language (TraML) format using the *TargetedFileConverter* tool, and then decoys were added using the *OpenSwathDecoyGenerator* tool with an identity threshold of 1 and a similarity level of 0.05 Da. The combined assay library in TraML format was used for downstream SWATH analysis.

### 4.8. SWATH-MS Data Acquisition and Library Validation

The library was validated using LC-MS/MS analysis on serum samples from PCa patients using a TripleTOF 6600 linked to a Micro HPLC system (Sciex, Warrington, UK). Serum samples from PCa patients were prepared using immunodepletion, protein concentration/buffer exchange, reduction/alkylation, and tryptic digestion. For analysis on the SWATH platform, dried tryptic peptides were reconstituted in a mixture of loading buffer (2% acetonitrile/0.1% formic acid), pepcalmix (10 fmol/μL, SCIEX, Warrington, UK), and index retention time (iRT) peptides (2X) (Biognosys AG, 8952 Schlieren, Switzerland) [16]. The analytical column was a YMC-Triart C18 12 nm, 3 m, 0.3 mm I.D. X 150 mm, 1/32” column (YMC Europe GmbH TA12S03-15HORU), whereas the trap column was a YMC-Triart C18 12 nm, S-3 m, 5 X 0.5 mm I.D., 1/32” column (YMC Europe GmbH TA12S03-15HORU) (YMC Europe GmbH TA12S03-E5JORU). The capacity of the sample pickup was set at 8 μL at a flow rate of 5 μL/min. The data were acquired using the 100 variable window method in conjunction with MS parameters; accumulation time was 0.249 s, the m/z range was 400–1250, the period was 119.98 min, the cycles were 2572, the delay time was 0 s, and the cycle time was 2.799 s [39].

Raw data files (.wiff) were searched against a combined purpose-built PCa serum library using openSWATH (version 2.0.0) [50]. PyProphet (version 0.18.3) [51] was used to score the peptide matches, and the results were aligned using the MSproteomicstools feature alignment [52]. Potential contaminants and decoy sequences were deleted before to conducting any analyses. An m-score threshold of 0.01 was utilised to annotate and filter the feature alignment file using the SWATH2Stats tool (Bioconductor packages release 3.11) [53]. The data were converted into a format that MSstats (Bioconductor packages release 3.11) [54] could interpret. The data were processed by selecting the ‘top3’ feature subset and then normalising the resulting information with the function “equalizeMedians.” The summaryMethod was set to “TMP,” the cutoffCensored to “minFeature,” and no missing data imputation was performed. In the acquired protein quants, all “NA” values were replaced with “0.” Random Forests (RF) were used to choose features, with data split 70:30 (training/testing) and ntree set to 1000 (Rstudio version 4.0.2). The gene ontology study was carried out using WebGestalt [55]. KEGG pathways and protein-protein interactions were investigated using the STRING database [56].

## 5. Conclusions

This study created a broad-spectrum PCa serum reference library by combining data from different LC technologies (micro-LC and nano-LC) and MS equipment. Additionally, PCa-related proteins were incorporated in silico to aid in developing biomarkers for PCa detection and surveillance. It is capable of processing clinical data acquired during SWATH-MS/DIA operations utilising a variety of high-resolution instruments. This library contains previously described blood and tissue biomarkers and a large number of proteins that are not identified in Pan-Human, PCa tissue, or cell-line reference libraries. This is the most comprehensive PCA-serum library accessible, with around 13,793 peptides and 1227 proteins. It identified 404 serum proteins in a validation dataset, indicating the library’s appropriateness for the purpose for which it was designed. It can be combined with other publicly accessible PCa-related tissue reference libraries to provide researchers with a complete picture of PCa proteome dynamics. With an increasing demand for novel biomarkers, this library provides an opportunity to identify clinically useful PCa biomarkers.

## Figures and Tables

**Table 1:** Details of the in silico (Disease), in-house built PCa spectral libraries acquired at Orbitrap Fusion Lumos Tribrid (Thermo Scientific, Waltham, MA, USA) mass spectrometer (in micro-flow mode) (Lumos), Sciex TripleTOF 6600 mass spectrometer in micro-flow mode (S-Micro) and nano-flow mode (S-Nano) and combined library (Combined-PCa) built after the integration all four, using an online tool, iSwathX (at protein FDR of 1%). S-Micro was used as base library and others added on in the process of integration.

Characteristics	Proteotypic	Proteotypic and Shared
Disease Library		
Proteins	346	346
Peptides	4503	4503
Precursors	7000	7000
Transitions	41,995	41,995
LUMOS library		
Proteins	561	760
Peptides	9176	9577
Precursors	13,263	13,891
Transitions	79,578	83,346
S-Micro Library		
Proteins	348	484
Peptides	5899	6210
Precursors	7931	8360
Transitions	47,586	50,160
S-Nano Library		
Proteins	508	712
Peptides	4110	4478
Precursors	5596	6104
Transitions	33,576	36,624
Combined Prostate Cancer Serum Library		
Proteins	950	1227
Peptides	13,265	13,793
Precursors	17,758	18,479
Transitions	110,346	114,684

## Data Availability

The mass spectrometry proteomics data have been deposited to the ProteomeXchange Consortium via the PRIDE [1] partner repository with the dataset identifier PXD028651. Reviewer account details are as follows: Username: reviewer_pxd028651@ebi.ac.uk and Password: 4kYGKQF6.

## Supplementary Materials

The following are available online at https://www.mdpi.com/article/10.3390/cancers13215580/s1, Figure S1: In-gel fractionation of pooled PCa and healthy controls serum samples loaded to a 10-well Bolt NuPAGE 4–12% Bis-Tris gel in MES Buffer and run at 200 Volts for 22 min. Followed by staining gel fractions were divided into 10 horizontal strips of equal sizes for further processing. Well 2 and 3 contained (70 µL each) pooled serum samples from 380 PCa patients, well 5 contained SeeBlue™ Plus2 Pre-stained Protein Standard, well 7 and 8 contained (70 µL each) pooled serum samples from 134 healthy men used as control. The band size of each markers’ band is given in parallel (in KDa), Figure S2: Distribution of peptide length (red dotted line represents amino acid cut-off of 15), peptides per protein, product charge (m/z) and precursor charge (m/z) for each consensus library used in this study, Figure S3: An outline of protein-protein interactions between proteins specific to the combined-PCa serum library when compared against the Pan-Human library based on a STRING database analysis, Table S1: Demographic and clinical characteristics of PCa patient cohort and healthy male volunteers used for generation of spectral library, Table S2: List of proteotypic/non-shared proteins identified across in silico disease library and in-house built spectral libraries acquired on Orbitrap Fusion Lumos Tribrid and Sciex TOF 6600 mass spectrometer coupled with micro and nano LC columns, Table S3: Tissue specificity of 154 proteins unique to the combined PCa serum spectral library when compared against the Pan-Human library, Table S4: Overlap of the total protein count of our combined serum PCa serum spectral library and PCa spectral libraries previously published from PCa tissue and cell lines samples (Liu et al.; Garrido-Rodriguez et al.), Table S5: To identify proteins associated with tumours or tumorigenesis, we compared a list of 569 genes from the catalogue of somatic mutations in cancer (COSMIC) database acquired from the Human Protein Atlas to a list of genes (acquired from Uniprot IDs) from our combined library, Table S6: Clinical characteristics of PCa patient cancer validation cohort of pre and post treatment radiotherapy samples used for validation of spectral library, Table S7: Assay characteristics of validation cohort following analysis against PCa serum spectral library, Table S8: Assay statistics across each validation sample after analysing them against PCa serum spectral library, Table S9. List of 17 proteins and their log 2 proteins intensity across pre and post radiotherapy samples used for validation of clinical application of PCa serum spectral library.
