# Supplementary material for: A Prostate Cancer Proteomics Database for SWATH-MS Based Protein Quantification
Source: Cancers (Basel). 2021 Nov 8;13(21):5580. doi: 10.3390/cancers13215580 (PMC8582933; doi:10.3390/cancers13215580)
Supplement: Supplementary file 1 [file cancers-13-05580-s001.zip › cancers-1408698-supplementary.pdf]

cancers

MDPI

Supplementary Material: A Prostate Cancer Proteomics Database for SWATH-MS based Protein Quantification

Ammara Muazzam, Davide Chiasserini, Janet Kelsall, Nophar Geifman, Anthony D. Whetton and Paul A. Townsend

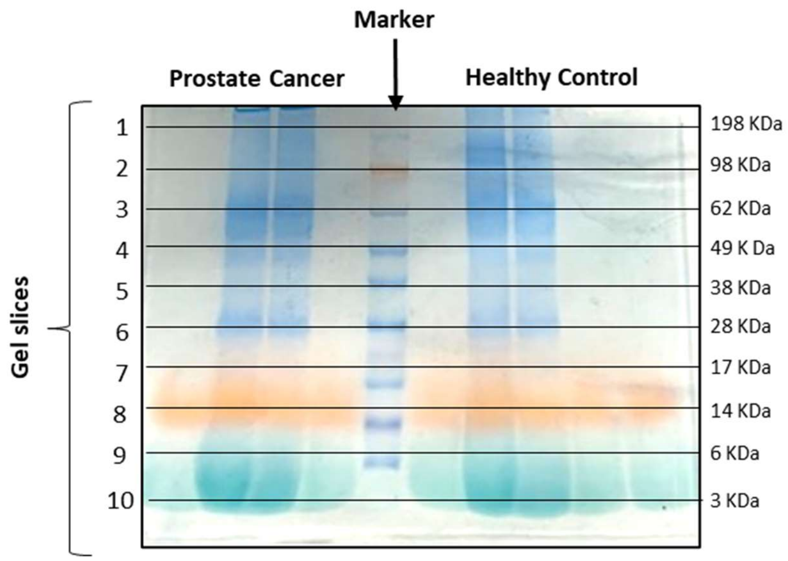

Figure S1. In-gel fractionation of pooled PCa and healthy controls serum samples loaded to a 10-well Bolt NuPAGE 4-12% Bis-Tris gel in MES Buffer and run at 200 Volts for 22 mins. Followed by staining gel fractions were divided into 10 horizontal strips of equal sizes for further processing. Well 2 and 3 contained (70 µl each) pooled serum samples from 380 PCa patients, well 5 contained SeeBlue™ Plus2 Pre-stained Protein Standard marker, well 7 and 8 contained (70 µl each) pooled serum samples from 134 healthy men used as control. The band size of each markers' band is given in parallel (in KDa).

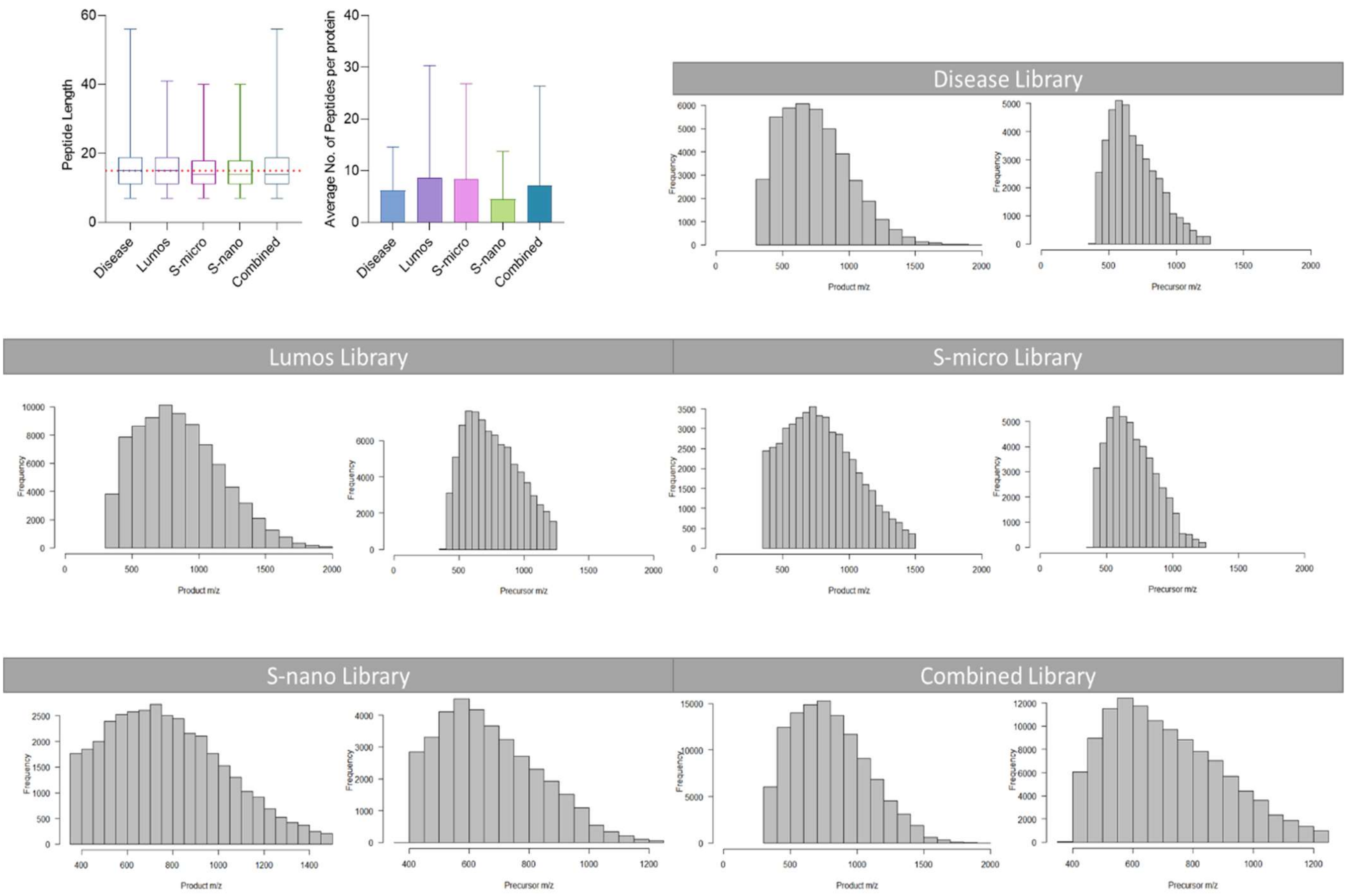

Figure S2. Distribution of peptide length (red dotted line represents amino acid cut-off of 15), peptides per protein, product charge (m/z) and precursor charge (m/z) for each consensus library used in this study.

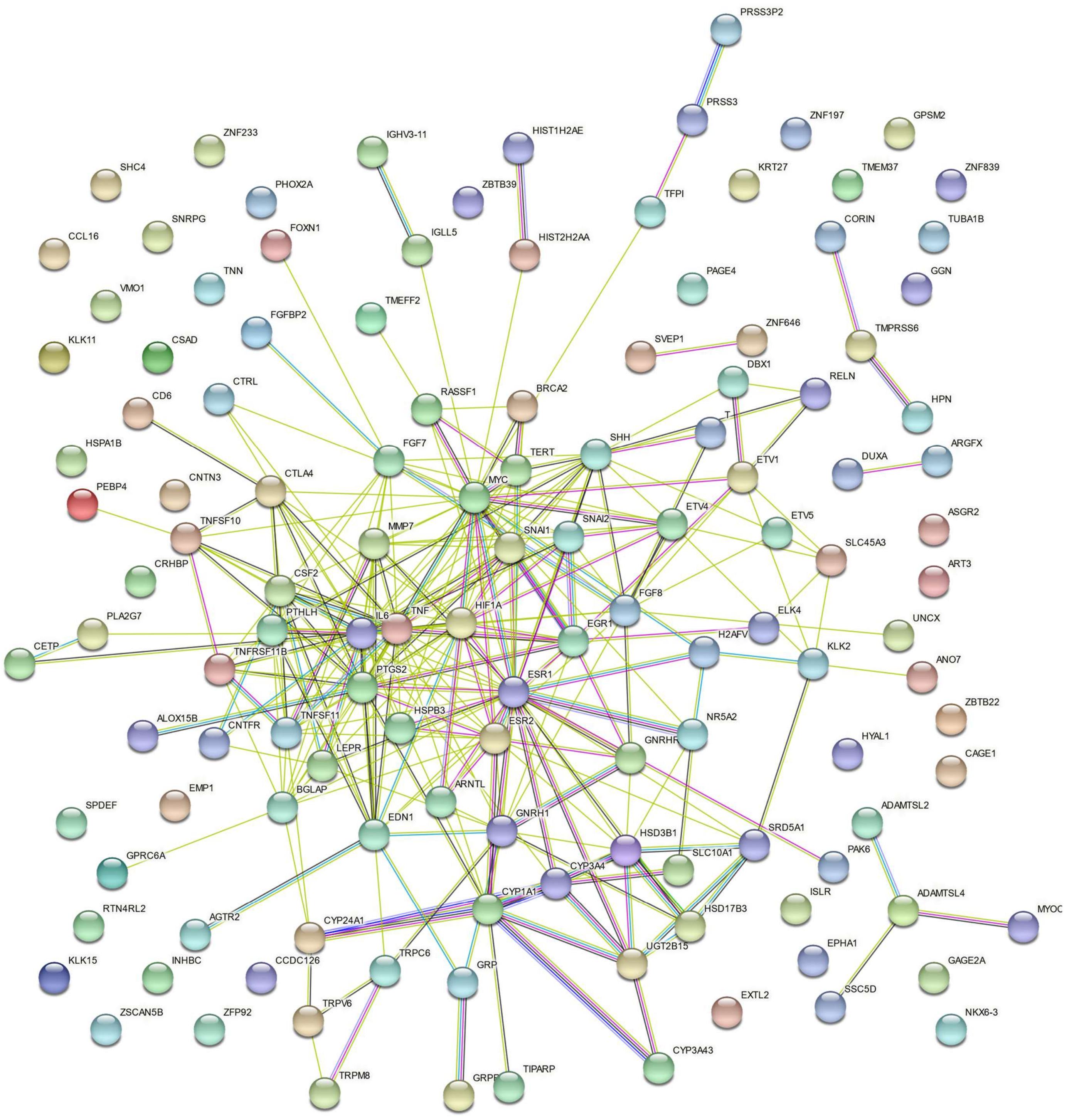

Figure S3. An outline of protein-protein interactions between proteins specific to the combined-PCa serum library when compared against the Pan-human library based on a STRING database analysis.
